# Monkeypox in Europe and beyond – tackling a neglected disease together

**DOI:** 10.2807/1560-7917.ES.2022.27.24.2200482

**Published:** 2022-06-16

**Authors:** Hans Kluge, Andrea Ammon

**Affiliations:** 1 World Health Organization Regional Office for Europe (WHO/Europe), Copenhagen, Denmark; 2 European Centre for Disease Prevention and Control (ECDC), Stockholm, Sweden

**Keywords:** monkeypox, monkeypox virus (MPXV), public health, epidemiology, research

Europe is currently at the epicentre of the largest and geographically most widespread monkeypox outbreak ever reported outside of endemic areas in western and central Africa. Over the past month, our learning curve of this well recognised but largely neglected infection has been steep. The response to this outbreak presents both new but also familiar challenges together with considerable uncertainty. One key challenge is access to diagnostics (particularly if scaling up is required) and another is that vaccines and therapeutics are limited in most settings. What is certain, however, is that the tremendous ability of our two organisations, the European Centre for Disease Prevention and Control (ECDC) and the World Health Organization Regional Office for Europe (WHO/Europe), to work together for better health for all the people across Europe during the coronavirus disease (COVID-19) pandemic will need to be leveraged and strengthened as we respond to this new cross-border health threat.

Across all Europe we are acting and will continue to act quickly, together, to rapidly understand, to respond to and to control this fast-evolving situation. We commit to doing so for all people, with combined technical high quality and efficiency, basing ourselves on principles of equity and no stigma or discrimination.

The first case of monkeypox not linked to travel to endemic countries was reported in the United Kingdom (UK) on 13 May 2022 [1], and only about a month later, as at 15 June, 25 countries across Europe have reported a total of 1,654 cases to the ECDC and WHO/Europe [2]. The situation is evolving rapidly, and the largest number of cases have so far been detected in the UK, Spain, Germany and Portugal while numbers also increase fast in some other European countries.

Last week’s and this week’s editions of *Eurosurveillance* included several rapid communications presenting early results from the outbreak investigations so far. Data from the Netherlands indicate that the mean incubation period for cases identified in the Netherlands up to 31 May is 8.5 days (5th to 95th percentiles: 4.2 to 17.3), providing additional evidence for the current recommendations for monitoring of contacts for 21 days [3]. Where data were available, all of the cases reported in the UK (except a family cluster) [1] and Portugal [4], have been identified among men. Many of the cases have been detected among adults, with a median age of 38 years in the UK and 33 years in Portugal. Over 83% of cases in the UK and all but one in Portugal were among self-identified gay, bisexual or other men who have sex with men. Although the disease has been mild in general, three of 27 individuals were hospitalised in Portugal. There is also concern about superimposed bacterial infections of lesions, such as described in the case reported from Australia [5].

We know from the literature that monkeypox has previously led to complications [6,7], including blindness and deforming scars. We also know that based on data mostly from outbreaks in western and central Africa, disease may be more severe among young children. Spread among younger age groups would therefore be of concern [6]. It is possible that other groups may also have severe outcomes from monkeypox, particularly pregnant women and people who are immunocompromised.

Even as new patients present every day, investigations into the early cases show that the outbreak in Europe was certainly underway as early as mid-April and highly likely earlier [1], based on early genomic data [8]. Strong surveillance and diagnostic systems in several European countries, along with swift cross-border information-sharing mechanisms across Europe, such as the ECDC EpiPulse system, the European Early Warning and Response System and the timely reporting under the International Health Regulations (IHR, 2005) ensured that the outbreak was rapidly reported, and information shared with concerned partners worldwide.

Control of the outbreak, however, remains challenging. The atypical clinical presentation of monkeypox during this outbreak, coupled with the lack of travel history may have delayed detection in the early phases. Many – but not all cases – report multiple and sometimes anonymous sexual partners as well as participation in various large events or parties. Identifying sexual partners and notifying them is therefore often difficult as reported in the UK, where only 22 of 78 sexual partners had names or contact details reported.

We must remember, however, as we have seen from previous outbreaks, that the monkeypox virus does not discriminate based on sexual orientation or gender identity. Rapid, amplified transmission has occurred in the context of the recent lifting of pandemic restrictions on international travel and events. The potential for further transmission in Europe and elsewhere over the summer is high. Over the coming months, several of the festivals and large parties planned provide further contexts where amplification may occur. But most of all, they also provide powerful opportunities to engage to raise awareness and strengthen individual and community protection. Cases detected in the UK reported the use of geospatial dating apps, and many of these providers have readily responded to the outbreak by providing targeted public health messages and directing users to resources from health authorities [9,10]. In the coming months, they may play a key role in providing information to their users, potentially in the context of mass gatherings. Many gay and bisexual communities have high awareness and rapid health-seeking behaviour when it comes to their and their communities’ sexual health; continued engagement with communities will therefore be essential to control this outbreak.

Currently, our common goal is to curb this outbreak by stopping human-to-human transmission across Europe and we are aware of the challenge. For an effective response to monkeypox in Europe we need a significant and urgent reduction in onward transmission through clear risk communication and community-led action to raise awareness to present to clinical services, to strengthen case detection and isolation during the infectious period, and effective contact tracing and monitoring.

Responding to monkeypox will need to be built up from the most affected communities. It will be important to actively engage community groups and leaders and civil society organisations to increase awareness and share information on how people can reduce their risk of exposure and rapidly identify early symptoms. The role of ECDC and WHO/Europe is to shape the environment, in such a way that conditions for an effective response are met. We believe there are several critical requirements for an effective response in Europe and our organisations will act together within their individual mandates to achieve them (Box).

Box **Critical elements for an effective response by ECDC and WHO/Europe to the monkeypox outbreak in Europe**

- **Ensuring our response tackles stigma and discrimination**

Throughout the pandemic, we have also seen how amplified misinformation can lead to negative public health outcomes. Monkeypox, which is not a well-known disease in Europe, has already led to misinformation and disinformation being manifested across social media and other platforms. ECDC and WHO/Europe are working to counter this through shared engagement with civil society organisations to listen, and co-create appropriate, practical and factual information for the general population and for priority groups or communities [11].

- **Closing outstanding knowledge gaps**

A number of unknowns remain around the emergence of monkeypox in Europe and beyond that will require close collaboration together with a range of partners – public health authorities, academia, clinicians and affected communities. These unknowns relate to issues such as the risk of spread in different population groups, the mechanism of transmission and the effectiveness of interventions.

- **High quality in our guidance and recommendations**

The strong ties between ECDC and WHO/Europe and our common stakeholders mean we deliver better when we speak with one voice. We are facing an atypical outbreak of a neglected disease and the response will need us to issue, and regularly update, guidance to our stakeholders. ECDC and WHO/Europe have already issued joint guidance on risk communication and community engagement that provides our common approach to what may be the most important public health intervention at this stage of the outbreak [12]. We also issued joint advice on mass gatherings, supporting public health authorities, organisers and participating communities to leverage these events to share accurate, practical and targeted information with participants [13].

- **Synergy through the complementary mandates of ECDC and WHO/Europe**

As two organisations with overlapping but separate mandates, we can leverage our comparative advantages to do more together. We will use this response to further join up our response systems, make better use of our combined knowledge and expertise, and remove the need for countries to duplicate actions. We have applied this principle throughout the COVID-19 pandemic, and across a number of other recent health threats and we will build on these successes for the monkeypox response. This means that where needed, we will develop and use shared data platforms, produce joint epidemiological reports, undertake combined regional surveys, and deploy synergistic support to countries.

COVID-19: coronavirus disease; ECDC: European Centre for Disease Prevention and Control; WHO/Europe: World Health Organization Regional Office for Europe.

Monkeypox in Europe is a cross-border phenomenon, requiring concerted action in the spirit of pan-European unity, transcending geopolitical divides to aim to contain the outbreak. Indeed, united action for better health in Europe has never been as critical as today. The monkeypox outbreak presents many unknowns including around the transmission dynamics of the virus, the epidemiology and clinical characteristics of the disease, aspects related to One Health, most effective methods for community engagement and countermeasures for managing the disease. Europe has now the responsibility to act collectively and promote the research to answer these questions, and to convert that knowledge into immediate and concrete action, for the benefit of our entire region, leaving no one behind.
